# Prognostic value of a hypoxia-related microRNA signature in patients with colorectal cancer

**DOI:** 10.18632/aging.102228

**Published:** 2020-01-11

**Authors:** Yongmei Yang, Ailin Qu, Qi Wu, Xin Zhang, Lili Wang, Chen Li, Zhaogang Dong, Lutao Du, Chuanxin Wang

**Affiliations:** 1 Department of Clinical Laboratory, Qilu Hospital, Shandong University, Jinan 250012, Shandong Province, China; 2 Key Laboratory of Tumor Marker Translational Medicine, Shandong Provincial Medicine and Health, Jinan 250012, Shandong Province, China; 3 Department of Clinical Laboratory, The Second Hospital of Shandong University, Jinan 250033, Shandong Province, China; 4 Department of Blood Transfusion, Qilu Hospital, Shandong University, Jinan 250012, Shandong Province, China

**Keywords:** colorectal cancer, hypoxia microRNA signature, prognosis, prediction, nomogram

## Abstract

Hypoxia has been particularly associated with poor prognosis in cancer patients. Recent studies have suggested that hypoxia-related miRNAs play a critical role in various cancers, including colorectal cancer (CRC). In the present study, we found 52 differentially expressed miRNAs in HT-29 cells under hypoxic conditions versus normoxic conditions by analyzing the profiles of miRNAs. Using Cox model, we developed a hypoxia-related miRNA signature consisting of four miRNAs, which could successfully discriminate high-risk patients in the Cancer Genome Atlas (TCGA) training cohort (n=381). The prognostic value of this signature was further confirmed in the TCGA testing cohort (n=190) and an independent validation cohort composed of formalin-fixed paraffin-embedded clinical CRC samples (n=220), respectively. Multivariable Cox regression and stratified survival analysis revealed this signature was an independent prognostic factor for CRC patients. Time-dependent receiver operating characteristic (ROC) analysis showed that the area under the curve (AUC) of this signature was significantly larger than that of any other clinical risk factors or single miRNA alone. A nomogram was constructed for clinical use, which incorporated both the miRNA signature and clinical risk factors and performed well in the calibration plots. Collectively, this novel hypoxia-related miRNA signature was an independent prognostic factor, and it possessed a stronger predictive power in identifying high-risk CRC patients than currently used clinicopathological features.

## INTRODUCTION

Colorectal cancer (CRC) is one of the most frequently diagnosed malignancies worldwide with substantial mortality [1]. The high mortality in CRC is largely attributed to its late detection and high recurrence rate, and the 5-year survival rate of CRC patients is highly dependent on the cancer stage at diagnosis. Accurate assessment of a patient’s prognosis is crucially important for treatment planning, as well as clear and effective communication between physicians and their patients. However, the most commonly used approach for predicting patient survival remains pathological staging according to the tumor-node-metastasis (TNM) classification system, and it provides only limited information for the clinical prognostication because even patients within the same stage exhibit huge variation in prognosis and treatment response [2–3]. Therefore, it is urgently necessary to develop robust prognostic biomarkers that can offer a superior prognostic clinical usefulness compared with traditional risk factors.

Hypoxia is a micro-environmental hallmark of poor-prognosis in most solid tumors. The hypoxic microenvironment plays a major role in controlling the phenotype and behavior of cancer cells, whereby oxygen is limited, and thus cancer cells improve their own genetic index for adaptation and survival under hypoxic stress [4]. Hypoxic tumors become more aggressive, invasive and resistant to chemo- and radiation- therapy. Therefore, tumor hypoxia and hypoxia-inducible factors (HIFs), its main mediators, are supposed to be therapeutic targets [5–7]. MicroRNAs (miRNAs) are small non-coding RNAs with regulatory functions, which play critical roles in human cancers. Previous studies have established the link between aberrant miRNA expression and hypoxia in various neoplasms [8–11]. In CRC, the cooperation between hypoxia and miRNAs can promote tumor progression through different mechanisms, leading to poor prognosis [12–17]. Our group has previously reported that the hypoxia-inducible miR-210 is an independent prognostic factor and contributes to metastasis in CRC [18]. These findings suggest that the hypoxia-induced dysregulation of miRNAs in cancer has potential prognostic implications. We hypothesized that a characteristic miRNA expression pattern was induced in regions with sustained and intermittent hypoxia in CRC, and the extent of hypoxic miRNA expression determined the aggressiveness, or in general, the prognosis. However, to date, there is no comprehensive analysis of prognostic biomarkers based on hypoxia-related miRNA expression profiles in CRC patients.

Previous studies have shown that the combination of a panel of multiple indicators, rather than just a single factor, will yield more powerful and accurate information in the clinical setting [4, 19–21]. In the present study, we conducted a systematic analysis and developed a novel hypoxia-related miRNA signature to predict individualized survival for CRC patients. We initially cultivated CRC cell lines under hypoxic conditions and screened hypoxia-related miRNAs by high-throughput sequencing (HTS). Next, we identified survival biomarkers using these miRNAs in The Cancer Genome Atlas (TCGA) database. Finally, the clinically relevant prognostic miRNA biomarkers were validated and incorporated into the prognostic nomogram for CRC patients.

## RESULTS

### Clinical characteristics of the enrolled participants

Table 1 showed the baseline clinical and pathological characteristics, which were similar among the training, internal testing and independent validation cohorts (all *P*>0.05).

**Table 1:** Baseline clinical and pathological characteristics in our study.

		**Training**	**Cohort**		**Test**	**Cohort**		**Validation**	**Cohort**		P*
		**Total**	**High risk**	**Low risk**	**Total**	**High risk**	**Low risk**	**Total**	**High risk**	**Low risk**	
Gender											0.081
	Female	184	25 (42.4%)	159 (49.4%)	80	11 (35.5%)	69 (43.4%)	117	15 (45.5%)	102 (54.5%)	
	Male	197	34 (57.6%)	163 (50.6%)	110	20 (64.5%)	90 (56.6%)	103	18 (54.5%)	85 (45.5%)	
Age^a^											0.943
	≥61	213	40 (67.8%)	173 (53.7%)	104	16 (51.6%)	88 (55.3%)	124	22 (66.7%)	102 (54.5%)	
	<61	168	19 (32.2%)	149 (46.3%)	86	15 (48.4%)	71 (44.7%)	96	11 (33.3%)	85 (45.5%)	
Tumor location											0.262
	Colon	276	40 (67.8%)	236 (73.3%)	142	18 (58.1%)	124 (78%)	149	18 (54.5%)	131 (70.1%)	
	Rectum	105	19 (32.2%)	86 (26.7%)	48	13 (41.9%)	35 (22.0%)	71	15 (45.5%)	56 (29.9%)	
Lymph node examined count											0.101
	12 or more	334	51 (89.5%)	283 (94.6%)	183	29 (93.5%)	154 (98.7%)	199	31 (93.9%)	168 (96%)	
	Less than 12	22	6 (10.5%)	16 (5.4%)	4	2 (6.5%)	2 (1.3%)	9	2 (6.1%)	7 (4.0%)	
CEA											0.697
	Abnormal	90	12 (38.7%)	78 (36.8%)	46	11 (50%)	35 (33%)	60	11 (50%)	49 (38.9%)	
	Normal	153	19 (61.3%)	134 (63.2%)	82	11 (50.0%)	71 (67.0%)	88	11 (50.0%)	77 (61.1%)	
stage											0.212
	Stage I	74	4 (7.1%)	70 (22.2%)	25	1 (3.4%)	24 (15.8%)	36	1 (3.1%)	35 (19.3%)	
	Stage II	138	19 (33.9%)	119 (37.8%)	63	12 (41.4%)	51 (33.6%)	90	23 (71.9%)	67 (37.0%)	
	Stage III	101	20 (35.7%)	81 (25.7%)	67	14 (48.3%)	53 (34.9%)	52	6 (18.8%)	46 (25.4%)	
	Stage IV	58	13 (23.2%)	45 (14.3%)	26	2 (6.9%)	24 (15.8%)	35	2 (6.2%)	33 (18.2%)	

P*: the difference between the training cohort, test cohort and validation cohort was examined using Pearson’s Chi-squared test.

^a^ The average age was 61.

### Identification of hypoxia-induced miRNAs from CRC cell sequencing data

**Figure 1:**
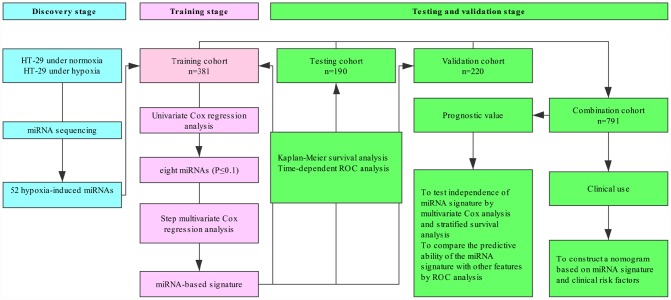
**Study flowchart.**

Our study was conducted in four stages as follows: discovery stage, training stage, testing stage and validation stage. A flowchart of the procedures was presented in Figure 1. To identify hypoxia-induced miRNAs, we cultured CRC cells (HT-29) under normoxic and hypoxic conditions for 48 h, and then performed HTS on these cells to identify the miRNAs with significantly altered expression. The expression of a miRNA was considered altered only if at least 50 counts were detected by HTS, together with fold change > 1.5 in its expression level between the hypoxic cells and normal ones. Based on these criteria, we found 52 differentially expressed miRNAs under hypoxic conditions (Supplementary Table 1). Moreover, they were therefore considered to be candidate hypoxia-induced miRNAs and subjected to subsequent analysis.

### Identification of the prognostic miRNAs from the training cohort

To single out the hypoxia-related prognostic miRNAs, 52 hypoxia-induced miRNAs were initially subjected to univariable Cox proportional hazards regression analysis in the training cohort. Among the 52 miRNAs, eight candidates with top statistical significance (*P*-value ≤0.1) were subsequently entered into a step multivariable Cox regression analysis (Supplementary Table 2). As a result, we trained a hypoxia-related prognostic signature consisting of four miRNAs (miR-375, miR-197, miR-26a and miR-210, Supplementary Table 3). Of these four miRNAs, miR-197, miR-210 and miR-26a with positive coefficient were risk factors owing to the close association between their high expression and poor survival of patients, whereas miR-375 was a protective factor. Supplementary Figure 1 listed the KEGG pathway analysis for these four miRNAs.

### Construction of a miRNA prognostic risk model and its predictability assessment in the training cohort

**Figure 2:**
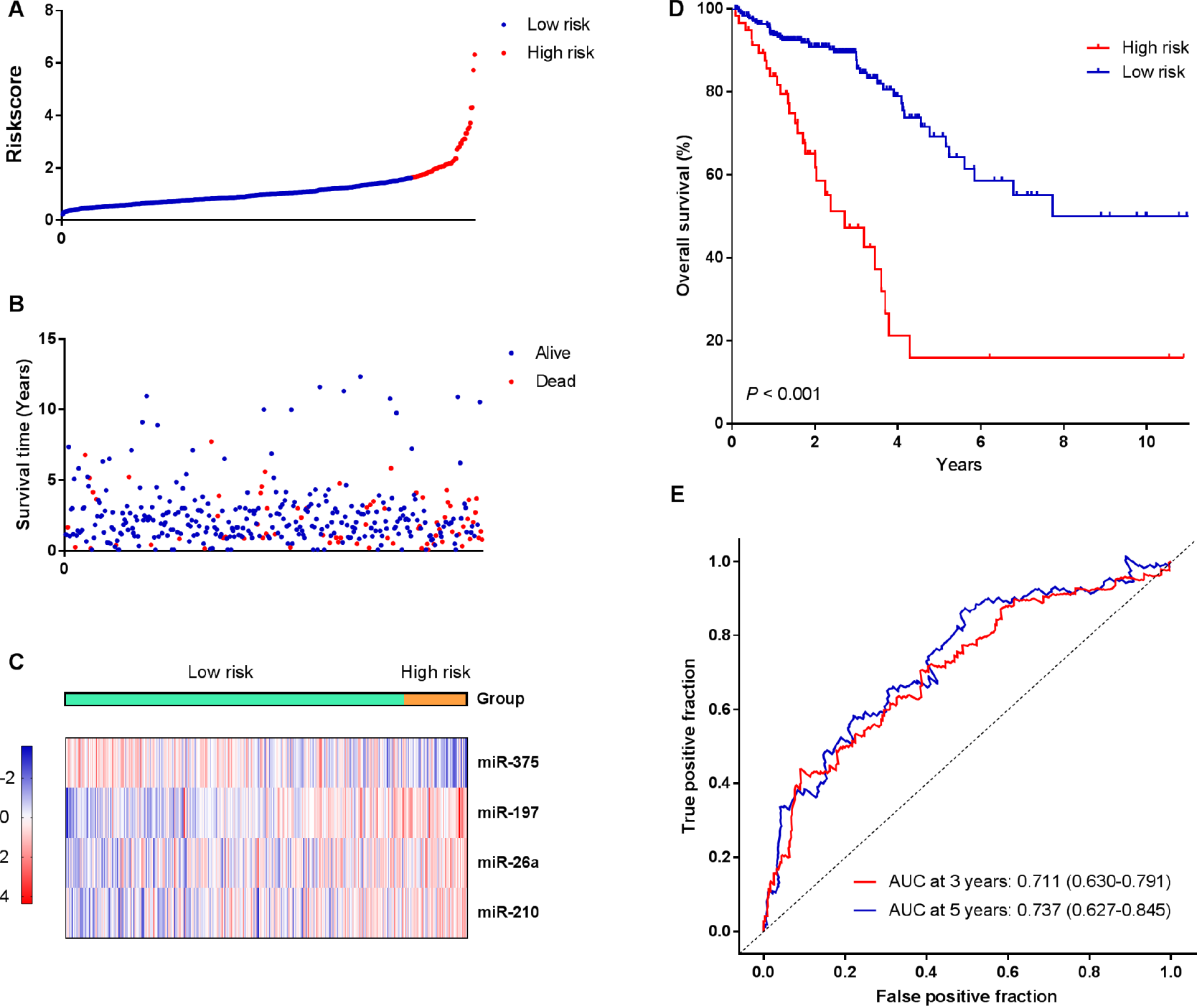
**Identification of a four-miRNA signature was significantly associated with OS of CRC patients in the training cohort.** (**A**–**C**) Risk score distribution, survival status, and miRNA expression patterns for patients in high-risk and low-risk groups by the miRNA signature. (**D**) Kaplan-Meier curve analysis of OS of CRC patients in high-risk and low-risk groups. (**E**) Time-dependent ROC curves analysis. We used AUCs at 3 and 5 years to assess the prognostic accuracy, and calculated P values using the log-rank test.

By using the regression coefficients of multivariable Cox regression model to weight the expression level of each miRNA in the hypoxia-related prognostic signature, we developed a risk score formula to predict the OS of CRC patients as follows: Risk score = (0.2113*miR-210) + (0.4688*miR-26a) + (0.4337*miR-197) + (-0.2266*miR-375). According to this formula, the risk score was calculated for each patient in the training cohort. Therefore, patients were dichotomized into high-risk group (n = 59) and low-risk group (n = 322) according to the optimum cutoff value generated by X-tile plots (Supplementary Figure 2). We ranked the risk scores of patients in the training cohort and analyzed their distributions in Figure 2A. The survival status of CRC patients was marked on the dot plot (Figure 2B). The results indicated that patients with higher risk scores generally had poorer OS than those with lower risk scores. The heatmap showed the expression pattern of prognostic miRNAs between the high-risk group and low-risk group (Figure 2C). For high-risk patients, three risk miRNAs (miR-197, miR-210 and miR-26a) were increased, and the protective miR-375 was decreased. On the contrary, these miRNAs displayed the opposite expression patterns in low-risk patients. Kaplan-Meier survival analysis (Figure 2D) demonstrated that high-risk patients had shorter OS than low-risk patients (log-rank test, *P*<0.001). We also assessed the prognostic ability of the miRNA signature using time-dependent ROC analysis at different time points. The results showed that the area under the ROC curve (AUC) for the four-miRNA prognostic model was 0.711 (95% CI: 0.630-0.791) at 3 years and 0.737 (95% CI: 0.627-0.845) at 5 years (Figure 2E). In the univariable Cox regression model of OS, the risk of death of high-risk group was increased by 4.241-fold compared with the low-risk group (95% CI:2.649-6.791, *P*< 0.001).

### Validation of the miRNA signature for OS prediction in the testing cohort

**Figure 3:**
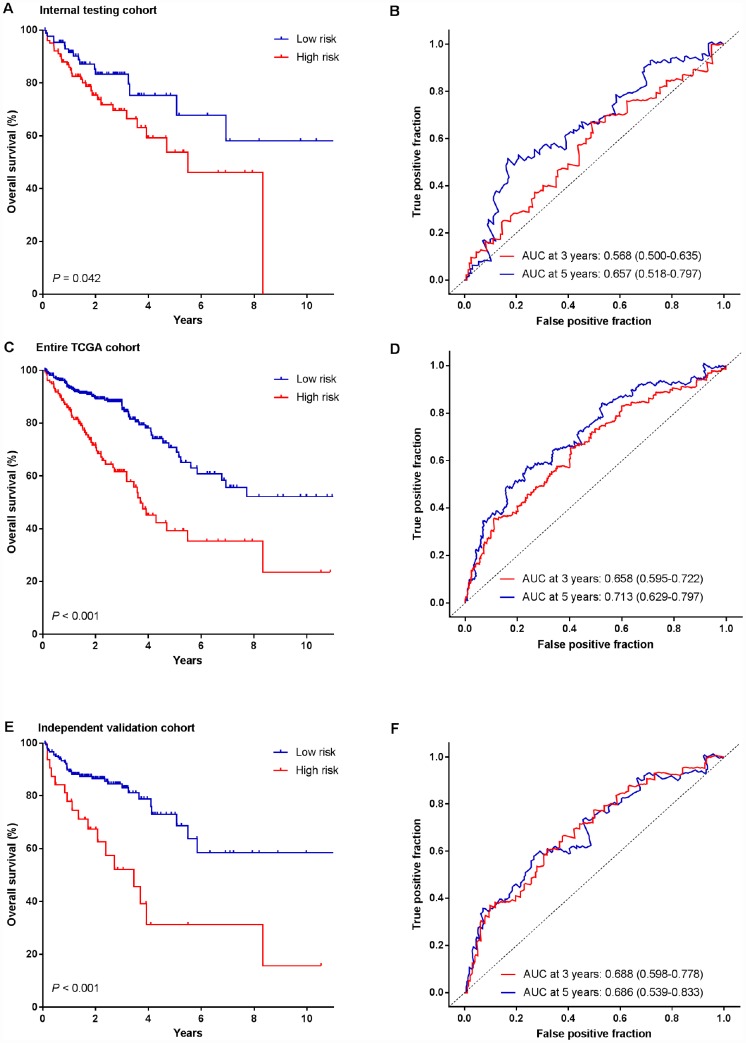
**Kaplan-Meier survival analysis and time-dependent ROC curves of the miRNA signature in the testing cohort, entire TCGA cohort and independent validation cohort.** (**A**, **B**) Internal testing cohort. (**C**, **D**) Entire TCGA cohort. (**E**, **F**) Independent validation cohort. We used AUCs at 3 and 5 years to assess prognostic accuracy, and calculated P values using the log-rank test.

To examine the robustness of the four-miRNA signature, the prognostic value of the signature was further tested using a testing cohort and an entire TCGA patient cohort. We used the same risk score formula obtained from the training cohort and computed the risk score for all patients in the testing cohort. Then the patients were classified into high-risk group and low-risk group using the same cutoff value obtained from the training cohort. We did the same survival analysis as in the training cohort. In line with the findings of training cohort, high-risk patients had shorter OS than low-risk patients in the testing cohort (Figure 3A, log-rank test, *P*=0.042). Time-dependent ROC analysis indicated that the AUC for the miRNA signature was 0.568 (95% CI: 0.500-0.635) at 3 years and 0.657 (95% CI: 0.518-0.797) at 5 years (Figure 3B), respectively. Risk score-based classification of the entire TCGA cohort also yielded similar results (log-rank test, *P* <0.001; AUC at 3 year: 0.658, 95% CI: 0.595-0.722; AUC at 5 year: 0.713, 95% CI: 0.629-0.797; Figure 3C and 3D).

### Revalidation of the four-miRNA signature for survival prediction by an independent validation cohort

To evaluate the translational potential of our miRNA signature in identifying high-risk patients, we deliberately examined its performance in FFPE tissues (independent validation cohort, n=220), which are routinely available in the clinical settings. Patients were classified into high-risk and low-risk groups according to their risk scores of the miRNA signature. Similarly, more patients with dead status fell into the high-risk group, in which the OS was shorter than that in the low-risk group (log-rank test, *P*<0.001, Figure 3E). Furthermore, the miRNA signature achieved an AUC of 0.688 (95% CI: 0.598-0.778) and 0.686 (95% CI: 0.539-0.833) at 3 and 5 years, respectively (Figure 3F).

### Prognostic value of the miRNA signature

**Figure 4:**
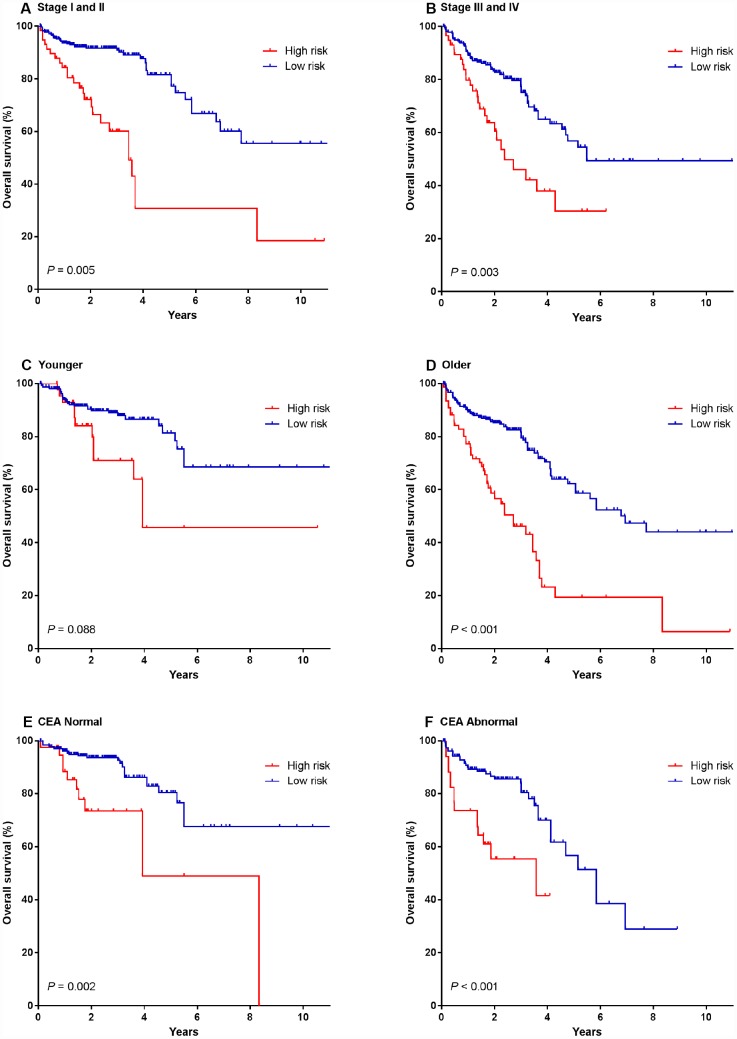
**Kaplan-Meier survival analysis according to the four-miRNA signature stratified by clinicopathological risk factors in all 791 CRC patients.** (**A**, **B**) TNM stage. (**C**, **D**) age. (**E**, **F**) preoperative CEA level. We calculated P values using the log-rank test.

To investigate whether the prognostic value of miRNA signature was independent of other clinicopathological variables, the univariable and multivariable Cox regression analyses were initially performed in the entire cohort consisting of 791 patients (combination of the training, testing and validation cohorts). We found that the risk score of the miRNA signature was significantly associated with the OS of patients even after adjustment by other clinical factors (Table 2). We also observed that patient’s age, preoperative CEA level and clinical stages were significant prognostic factors in CRC patients (all *P*<0.05). Therefore, data stratification analysis was introduced to determine the independence of our miRNA signature according to age, preoperative CEA level and clinical stages. Figure 4 illustrated that the survival curves of high-risk group situated below those of low-risk group in all subgroups. Log-rank tests showed that our miRNA signature was still a clinically and statistically significant prognostic factor in all subgroups, except for younger patients. In the subgroup of younger patients, our miRNA signature was a marginally significant prognostic factor (*P*=0.088).

**Table 2:** Univariate and multivariate Cox proportional hazards analysis of factors associated with OS in all 791 patients.

**Variables**	**Univariable analysis**			**Multivariable analysis**		
	**HR**	**95% CI**	***P*-value**	**HR**	**95% CI**	***P*-value**
Gender						
Male vs female	1.125	0.832-1.520	0.444			
Age						
Older vs. younger	2.126	1.520-2.973	0.000	3.204	1.910-5.374	0.000
Tumor location						
rectum vs. colon)	0.899	0.632-1.279	0.555			
MiRNA signature						
High vs low	3.007	2.188-4.133	0.000	2.806	1.724-4.566	0.000
Lymph node examined count					
12 or more vs. less than 12	0.747	0.423-1.321	0.316			
Preoperative CEA						
Abnormal vs. normal	2.379	1.558-3.630	0.000	2.166	1.392-3.371	0.001
Stage group						
Late group vs. early group	1.743	1.275-2.383	0.000	1.944	1.233-3.065	0.004

**Figure 5:**
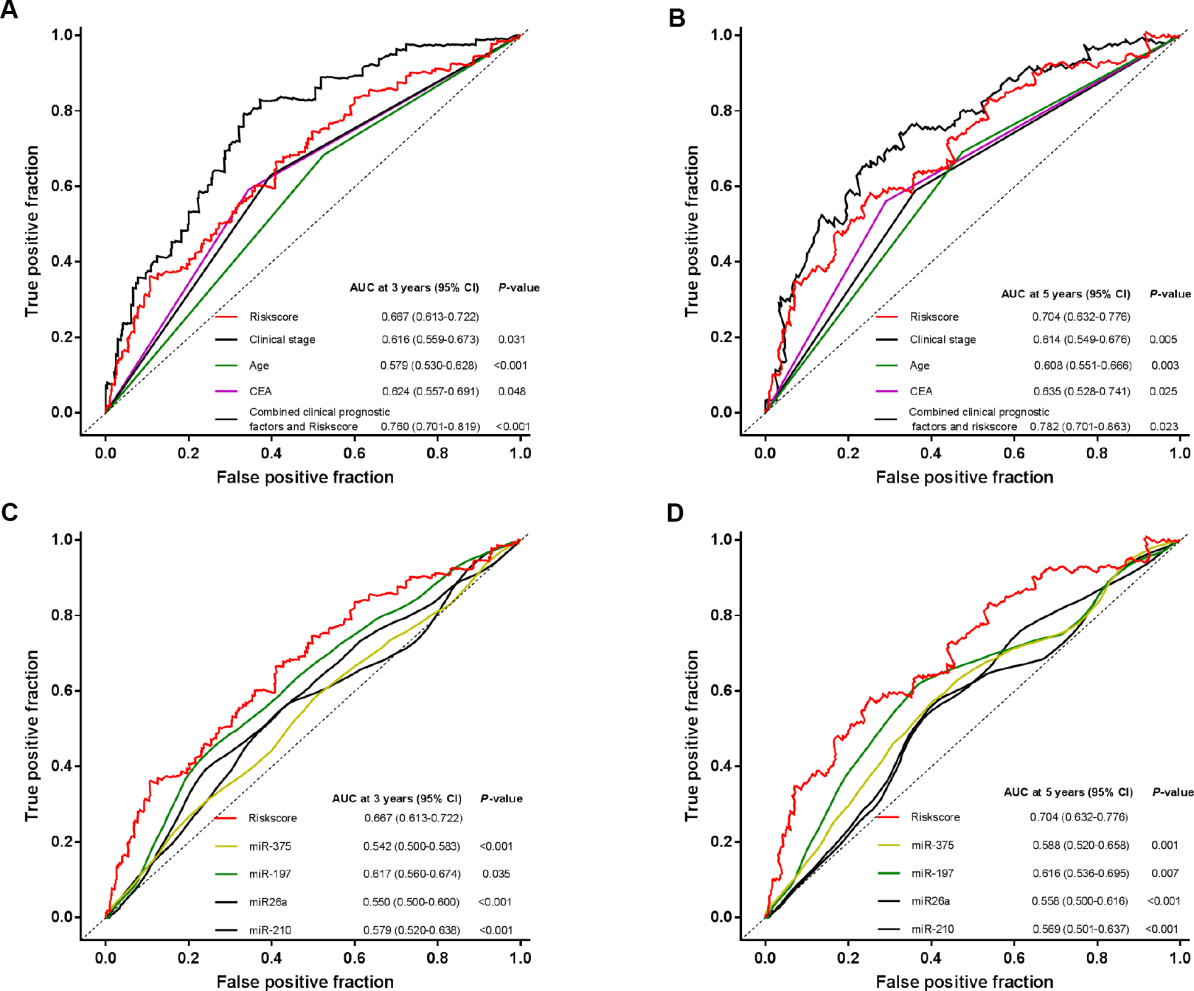
**Time-dependent ROC curves to compare the prognostic accuracy of the four-miRNA signature with clinicopathological risk factors and single miRNAs in all the 791 patients.** (**A**, **B**) Comparisons of the prognostic accuracy by the four-miRNA-based signature, age, preoperative CEA level, clinical stages, and combined clinicopathological prognostic factors and miRNA signature. (**C**, **D**) Comparisons of the prognostic accuracy by the four-miRNA-based signature, and miR-210, miR-375, miR-26a, miR-197. P values were from the comparisons of the AUC of the miRNA signature versus the AUC of other factors.

We also performed ROC analysis to compare the predictive ability of the miRNA signature with other features. Figure 5 showed that the four-miRNA risk score model possessed a stronger predictive power than other clinical risk factors (age, preoperative CEA level and clinical stages), or single miRNA alone (all *P*<0.05). When the risk score was combined with clinicopathological risk factors, significant difference was found between the combined model and the risk score (*P*<0.05). The results further confirmed the reliable predictive ability of our miRNA signature.

### Construction of a nomogram based on the miRNA signature

**Figure 6:**
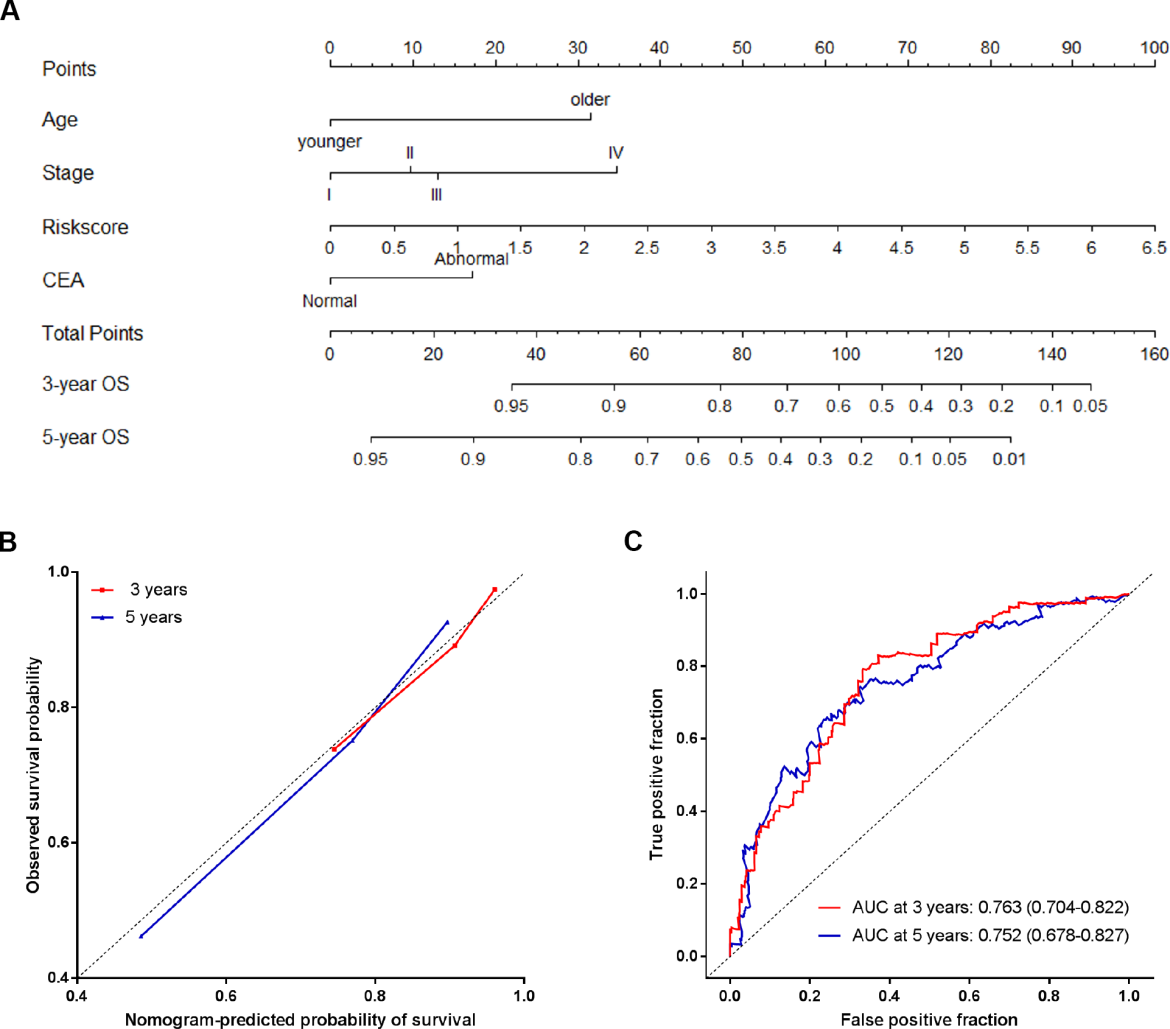
**The nomogram to predict probability of OS for CRC patients in all 791 patients.** (**A**) The nomogram for predicting proportion of patients with OS. (**B**) The calibration plots of the nomogram for the probability of OS at 3 and 5 years. (**C**) Time-dependent ROC based on the nomogram for survival probability. Nomogram-predicted probability of survival is plotted on the x-axis, and observed survival probability is plotted on the y-axis.

We constructed a nomogram to provide a quantitative method for clinicians to predict individual probability of survival, in which the miRNA signature was integrated with clinicopathological independent risk factors for survival (including age, tumor stage and preoperative CEA level) in CRC patients (Figure 6A). The bias-corrected lines of both 3 and 5 years in the calibration plot were very close to the ideal curve (the 45-degree line), indicating good agreements between prediction and observation (Figure 6B). The predictive accuracy of the nomogram was calculated through survival ROC analysis. The AUCs of nomogram at 3 and 5 years were 0.763 (95% CI: 0.704-0.822) and 0.752 (95% CI: 0.678-0.827), respectively, suggesting the favorable discrimination performance (Figure 6C).

## DISCUSSION

CRC is associated with a high morbidity and mortality rate. It is urgently necessary to identify new prognostic indicators in order to more accurately predict prognosis of patients in the era of precision medicine. In the present study, we firstly screened differentially expressed miRNAs related to hypoxia by miRNA sequencing. Then, we employed a large cohort of CRC patients from TCGA training cohort and trained a hypoxic signature of miRNAs (miR-197, miR-26a, miR-210 and miR-375) for predicting OS in CRC patients, followed by validation of its clinical value in the testing cohort. Finally, the clinical significance of this signature was reconfirmed by using an independent validation cohort from our hospital, with superior prognostic and predictive value compared with clinical risk factors.

Hypoxia is a major contributor to the failure of conventional cancer therapies in the tumor microenvironment. However, it is seldom considered in clinical settings, in part, due to the lack of good endogenous hypoxia markers [22]. The over-expression of HIF has been proved to be closely associated with prognosis, while HIF itself is not actually considered to be a hypoxia marker exclusively because it can be activated in various ways, including oncogene drive [23]. Therefore, we attempted to circumvent this limitation by developing a hypoxia-related miRNA signature for predicting prognosis in CRC patients.

MiRNAs are promising cancer biomarkers, which have great potential to be used in personalized medicine, and may be regulated by hypoxia in multiple human tumors. Several miRNAs are hypoxia sensors and thus termed hypoxia-responsive miRNAs or hypoxamiRs [8, 24–25]. In the current study, we initially found that 52 miRNAs were consistently differentially expressed in CRC cells cultured under the hypoxic conditions compared with normoxic conditions. Furthermore, we, for the first time, demonstrated a correlation between the altered expressions of four miRNAs under hypoxic conditions in vitro with those of tumors in TCGA datasets with available clinical data. To the best of our knowledge, this study was the first attempt ever made to comprehensively analyze the prognostic biomarkers based on hypoxic miRNA expression profiles in cancer patients.

Among the identified miRNAs, miR-197, miR-26a and miR-210 were risk factors, whereas miR-375 was a protective factor. Both high levels of risk factors and low levels of the protective factor were associated with a poor prognosis in CRC patients. We presumed that the four hypoxia-inducible miRNAs played critical roles in cancer progression and were prognostic indicators in CRC. Functional enrichment analysis of the KEGG signaling pathway showed the top 20 pathways involved, such as the MAPK signaling pathway, indicating these miRNAs served a critical role in the initiation and progression of CRC. Consistently, miR-26a has been proposed as an oncomir in the progression and invasion of CRC [26], which has also been shown to be up-regulated by hypoxia in glioblastoma multiforme, one of the most hypoxic tumors of the central nervous system [27]. Moreover, miR-210, the master hypoxamiR, generally exhibits oncogenic properties in most human solid tumors, including CRC [28]. However, miR-197-3p, another risk factor identified in this study, has been found to exhibit both oncogenic and tumor suppressive functions in different cancer types [29–30], and its hypoxia-regulated property was firstly reported here. MiR-375, the protective factor determined here, can inhibit autophagy and reduce viability of hepatocellular carcinoma cells under hypoxic conditions as previously reported [31]. These findings, together with our data, suggested the potential application of the four hypoxia- and survival-associated miRNAs as prognostic biomarkers in CRC patients. Meanwhile, more comprehensive and detailed studies are still required to assess the exact contribution of these miRNAs under hypoxic conditions in CRC.

In the current study, the combined index of the four miRNAs exhibited a significant association with prognosis in CRC patients. Multivariable analysis revealed that the four-miRNA signature predicted prognosis of CRC independently of traditional clinical risk factors. Further stratification analysis showed that this signature could reliably discriminate patients at high-risk from those at low-risk within all subgroups. Time-independent ROC analysis showed that it was superior to conventional TNM staging in predicting outcome of CRC patients. To improve the ability of prognostic prediction, we combined the four-miRNA signature with clinicopathological risk factors. The results indicated that there was a significant difference between the combined model and the miRNA signature. Similarly, a hypoxia-driven gene score has previously been developed, showing independent prognostic value in stage II and III colon cancer [32]. In this research, the hypoxia-related miRNA signature was firstly developed and successfully validated to predict prognosis of CRC patients.

Additionally, an ideal classifier for cancer prognostic prediction should be not only robust but also potentially feasible in available, archival samples. It has been reported that miRNAs are sufficiently stable in FFPE tissues, overcoming the barriers of sample collection and storage [33]. Therefore, this approach using FFPE specimens might be readily translated into clinical practice.

Nomograms are useful for the visualization of statistical models, graphical assessment of variable importance and the calculation of predicted values [34–35]. They have been widely used in predicting cancer risk, metastasis and therapeutic outcomes [19, 36–38]. Although nomograms are getting increasingly popular, few studies have created prognostic models using combinations of multiple miRNA biomarkers and clinical risk factors. Most recently, our group has successfully built a prognostic nomogram that integrates both a miRNA-based signature and clinical-related variables in gastric cancer [20]. In this study, based on the combination of the four-miRNA signature and independent clinicopathological variables, we established a nomogram model that could provide an individual prediction of prognosis in CRC patients. The calibration plot showed that the nomogram performed well, indicating good agreements between prediction and observation. The AUCs of nomogram at 3 and 5 years were 0.763 and 0.752, respectively, suggesting the favorable discrimination performance. Therefore, our nomogram might be a useful tool for patient counseling and personalized management for CRC patients.

Collectively, we demonstrated that the miRNA expression pattern of CRC cells under hypoxic conditions was significantly correlated with clinical parameters of CRC patients. Furthermore, a hypoxia-related miRNA signature was developed and proved to be an independent prognostic biomarker for prognosis prediction in CRC patients, which was superior to a model using only clinical risk factors. Moreover, this approach should be validated in large-scale multi-center clinical trials.

## MATERIALS AND METHODS

### Ethical statement

All human-related procedures were in accordance with the ethical standards of the Clinical Research Ethics Committee of Qilu Hospital, Shandong University and the Declaration of Helsinki. Written informed contents were obtained from all participants.

### Patients and clinical database

This study included patients from the publicly accessible dataset from TCGA and a clinical validation cohort of Qilu Hospital, Shandong University. In TCGA dataset, the miRNA sequencing data (containing expression data of 1,881 noted miRNAs) and corresponding clinicopathological information of CRC patients were downloaded from website (https://portal.gdc.cancer.gov). Patients with missing survival data and follow-up time less than 30 days were excluded. Consequently, a total 571 CRC patients were enrolled in the present study, which were randomly divided into a training cohort (n = 381, 2/3) and a testing cohort (n = 190, 1/3). In the clinical validation cohort, a total of 220 formalin-fixed paraffin-embedded (FFPE) specimens were collected from CRC patients who were treated in the Qilu Hospital, Shandong University (Jinan, China) between September 2007 and December 2012, according to the following criteria: (a) patients who underwent surgery with curative intent; (b) relevant clinical characteristics and follow-up data were available; (c) patients didn’t received preoperative therapy (radiotherapy, chemotherapy or chemoradiotherapy); and (d) patients didn’t simultaneously suffer from other tumor diseases. All samples were evaluated by two pathologists according to the 7^th^ edition of the American Joint Committee on Cancer TNM grading system. Relevant clinical information was collected from medical records.

### Cell culture and miRNA sequencing analysis

Human colorectal cell line HT-29 was purchased from the Type Culture Collection of the Chinese Academy of Sciences (Shanghai, China), and cells were maintained in DMEM supplemented with 10% fetal bovine serum (Gibco, Carlsbad, CA, USA) in a humidified incubator (5% CO_2_ at 37°C). HT-29 cells were initially cultured with 20% O_2_ for 24 h. After cells reached 60% confluence, cells were either placed under hypoxic conditions (2% O_2_, 5% CO_2_ at 37°C) or remained under normoxic conditions (20% O_2_) for another 48 h.

Total RNA was extracted from cells using Trizol reagent (Invitrogen, Carlsbad, CA) according to the manufacturer's instructions. Purity and integrity of RNA were quantified using both kaiaoK5500^®^Spectrophotometer (Kaiao, Beijing, China) and Agilent 2100 RNA Nano 6000 Assay Kit (Agilent Technologies, CA, USA). Total RNA was separated by 15% agarose gels to extract the small RNAs (18-30 nt). After precipitated by ethanol and centrifugal enrichment of small RNA sample, the library was prepared according to the method and process of Small RNA Sample Preparation Kit (Illumina, RS-200-0048). The qualified libraries were then sequenced on an Illumina Hiseq 2500 platform (Illumina, USA), and 50-bp single-end reads were generated.

To ensure the quality of data used in further analysis, the raw data were filtered out with Python scripts according the following criteria: (a) reads without 3’ adapter were removed; (b) reads without insert fragment were removed; (c) reads with too much poly A/T were removed; (d) reads with length out of certain range were removed; (e) the low-quality reads were removed; (f) the reads containing N base more than 5% for total bases were removed. The clean data were mapped to the reference genome by Bowtie1.0.1. Subsequently, mapping reads were matched to miRBase ((Release 21) to identify miRNAs. The quantitation of miRNA expression levels was used by count and RPM (reads per million total reads). Differential miRNA expression analysis was carried out using DEGseq. The entire procedure was performed in Annoroad Gene Technology Corporation (Beijing, China).

### RT-qPCR analysis of miRNA expression

Total RNA was extracted from 10-μm-thick FFPE specimens using the miRNA Isolation Kits (Bioteke, Beijing, China). All the manipulations of the RNA were carried out under RNase-free conditions, and cDNA was synthesized using miRNA-specific Bugle-Loop primers (Ribobio, Guangzhou, China) and the M-MLV RT kit according to the manufacturer’s instructions (Invitrogen, Carlsbad, CA, USA). miRNA expression was assessed by qRT-PCR using ABI PRISM 7500 Sequence Detection System (Applied Biosystems, Foster City, CA). The relative expressions of miRNAs were determined using the 2^−ddCT^ method with the U6 small nuclear RNA (U6) as the housekeeping gene. The normalized values were further log2 transformed. All the primers for miRNAs used in this study were synthesized by Ribobio.

### Study procedures

Our study was conducted in four stages as follows: discovery stage, training stage, testing stage and validation stage. A flowchart of the procedures was presented in Figure 1. In the discovery stage, HT-29 cells cultivated under normoxic and hypoxic conditions were subjected to miRNA sequencing to identify the miRNAs with significantly altered expression. In the training stage, the candidate hypoxia-induced miRNAs were initially subjected to univariable Cox proportional hazard regression analysis to examine the association between miRNA expression and overall survival (OS) in the training cohort. The miRNAs with top statistical significance (*P*-value≤0.1) were subsequently entered into a step multivariable Cox regression analysis with the Akaike information criterion (AIC) employed as the stopping rule to train a hypoxia-induced miRNA signature. An individual’s risk score model for each patient was built to predict prognosis of CRC patients using selected miRNA expression, weighted by the multivariable Cox regression coefficients as follows: Risks core = ∑_i_ coefficient (miRNA_i_) × expression (miRNA_i_). Using the optimum cut-off value obtained from X-tile plots (X-tile, version 3.6.1; Yale University School of Medicine, New Haven, CT, USA) in the training cohort, CRC patients could be sorted into high-risk group and low-risk group. The Kaplan-Meier method was used to draw survival curves of both high-risk group and low-risk group, which were compared by log-rank tests. The time-dependent receiver operating characteristic (ROC) curve were used to assess the prognostic performance of miRNA-based signature. In the testing stage, the same risk score formula obtained from the training cohort was used to compute the risk score for all patients in the testing cohort. Then the patients were classified into high-risk group and low-risk group using the same cutoff value obtained from the training cohort. The same survival analysis (Kaplan-Meier curve and time-dependent ROC curve) was performed in the training stage to examine the prognostic value of the miRNA signature in the testing cohort. In the validation stage, the performance of the miRNA signature in the independent validation cohort was determined, which consisted of 220 FFPE tissues from CRC patients.

### Statistical analysis

All the statistical analyses were performed with R software (version 3.4.2; http://www.Rproject.org). A *P*<0.05 was considered as significant. Categorical variables were presented as numbers (%) and the difference between the training cohort, test cohort and validation cohort was examined using Pearson’s Chi-squared test. A Cox proportional hazard regression model was applied for the univariable analysis and multivariable analysis of prognostic factors. We adopted the ‘survivalROC’ package for time-dependent ROC analysis, and the ‘bootstrap’ method (‘boot’ package) was used to examine the significance of differences between the ROC curves [39]. The regression coefficients in multivariable Cox regression model were used to generate the nomogram. Calibration plot and ROC curve were used to evaluate the performance of nomogram (“rms” package). KEGG pathway enrichment analysis was performed by “clusterProfiler” package.

## Supplementary Material

Supplementary Figures

Supplementary Tables
