# Supplementary material for: Prognostic value of a hypoxia-related microRNA signature in patients with colorectal cancer
Source: Aging (Albany NY). 2020 Jan 11;12(1):35–52. doi: 10.18632/aging.102228 (PMC6977676; doi:10.18632/aging.102228)
Supplement: Supplementary Figures [file aging-12-102228-s002..pdf]

SUPPLEMENTARY FIGURES

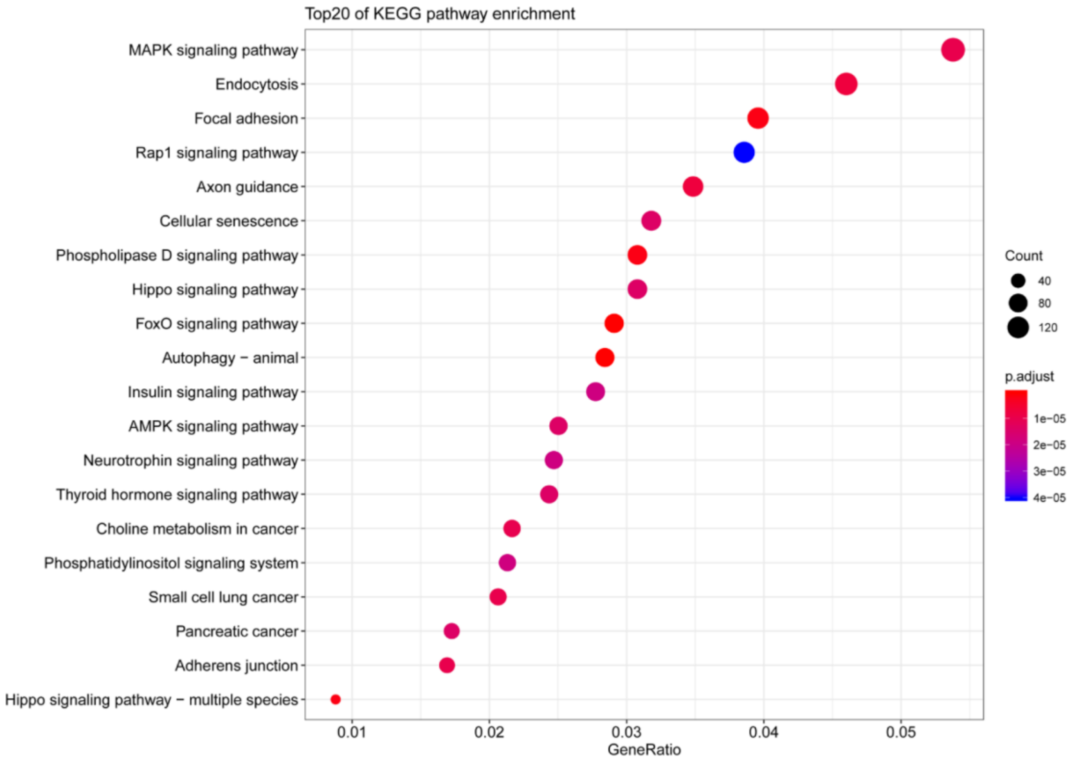

Supplementary Figure 1. The KEGG pathway analysis for the four miRNAs and the top 20 pathways involved.

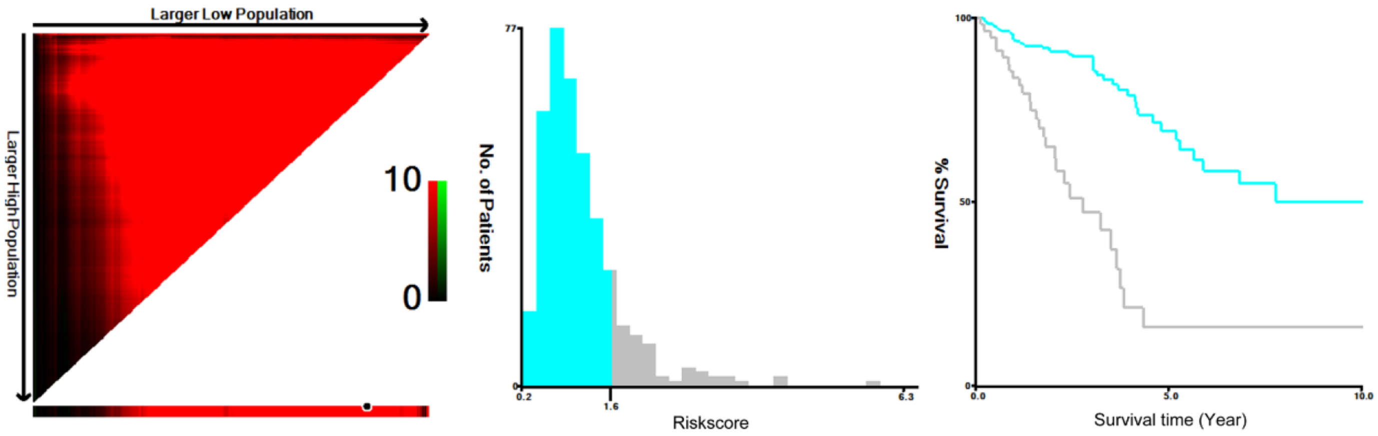

Supplementary Figure 2. X-tile plots of the four-miRNA signature and the risk score in the training cohort.
