# Supplementary material for: Prognostic value of a hypoxia-related microRNA signature in patients with colorectal cancer
Source: Aging (Albany NY). 2020 Jan 11;12(1):35–52. doi: 10.18632/aging.102228 (PMC6977676; doi:10.18632/aging.102228)
Supplement: Supplementary Tables [file aging-12-102228-s001..pdf]

SUPPLEMENTARY TABLES

Supplementary Table 1. Differentially expressed miRNAs in CRC cell line under hypoxia condition.

miRNA	Hypoxic	Normoxic	FoldChange
hsa-miR-210-5p	168.84	6.04	27.96
hsa-miR-10a-5p	11613980.05	791516.09	14.67
hsa-miR-652-5p	92.19	6.79	13.58
hsa-miR-210-3p	3511.05	316.69	11.09
hsa-miR-99b-5p	705702.39	65443.74	10.78
hsa-miR-192-5p	2463861.74	301687.42	8.17
hsa-miR-141-3p	307936.51	79991.06	3.85
hsa-miR-193b-3p	2890.80	818.13	3.53
hsa-miR-215-5p	1123.12	321.73	3.49
hsa-miR-941	30086.88	8867.20	3.39
hsa-miR-16-5p	344796.86	102582.72	3.36
hsa-miR-331-3p	346.68	107.35	3.23
hsa-miR-550a-3p	525.36	168.81	3.11
hsa-miR-21-5p	697516.85	224707.76	3.10
hsa-miR-30d-5p	315001.34	103601.07	3.04
hsa-miR-183-5p	45895.05	15311.03	3.00
hsa-miR-30b-5p	11502.89	3885.23	2.96
hsa-miR-31-3p	620.51	209.97	2.96
hsa-miR-197-3p	1173.87	404.11	2.90
hsa-miR-30e-5p	94605.57	32778.45	2.89
hsa-miR-26a-5p	433208.51	156066.86	2.78
hsa-miR-125a-5p	138827.40	51528.92	2.69
hsa-miR-18a-5p	1406.64	529.91	2.65
hsa-miR-21-3p	45518.13	18019.37	2.53
hsa-miR-23a-3p	8189.98	3324.28	2.46
hsa-miR-151a-3p	167776.37	72283.07	2.32
hsa-miR-200b-3p	199725.93	88461.86	2.26
hsa-miR-582-5p	423.02	193.33	2.19
hsa-miR-27a-3p	26596.73	12251.40	2.17
hsa-miR-27b-3p	124546.09	58784.65	2.12
hsa-miR-26b-5p	65556.75	31022.98	2.11
hsa-miR-1286	172.43	82.54	2.09
hsa-miR-181c-5p	3003.48	1448.35	2.07
hsa-miR-182-5p	245423.77	118622.92	2.07
hsa-miR-200a-3p	115773.85	57347.64	2.02
hsa-miR-200c-3p	24688.31	12247.79	2.02
hsa-miR-22-3p	168881.58	94448.32	1.79
hsa-miR-301a-3p	12705.48	7131.96	1.78
hsa-miR-20a-5p	5967.14	3422.89	1.74
hsa-let-7d-3p	792.26	464.23	1.71
hsa-miR-23b-3p	2316.65	1368.87	1.69
hsa-miR-574-3p	456.61	270.67	1.69
hsa-miR-151a-5p	85234.44	51454.79	1.66
hsa-let-7a-5p	83266.25	50540.95	1.65
hsa-miR-98-5p	9186.13	5778.80	1.59
hsa-miR-340-5p	4452.07	2851.35	1.56
hsa-miR-374a-3p	4720.77	3036.75	1.55

hsa-miR-148b-3p	4691.86	3022.09	1.55
hsa-miR-181a-5p	250152.58	162245.75	1.54
hsa-miR-3176	494.98	322.52	1.53
hsa-miR-375	31097.71	20541.12	1.51
hsa-miR-301b-3p	3619.22	2405.09	1.50

Supplementary Table 2. Univariable Cox proportional hazards regression analysis results in the training cohort.

miRNAs	HR	*P-value*
hsa-mir-26b	1.70	0.00
hsa-mir-197	1.57	0.01
hsa-mir-375	0.82	0.02
hsa-mir-98	1.39	0.06
hsa-mir-193b	1.25	0.07
hsa-mir-26a-2	1.35	0.09
hsa-mir-210	1.13	0.10
hsa-mir-26a-1	1.34	0.10

Supplementary Table 3. The results of step multivariable Cox regression analysis.

miRNA	Coefficients	HR	P-value
miR-197	0.43	1.54	0.02
miR-26a	0.47	1.60	0.01
miR-210	0.21	1.24	0.01
miR-375	-0.23	0.80	0.01
